# Online assessment of ALS functional rating scale compares well to in-clinic evaluation: A prospective trial

**DOI:** 10.3109/17482968.2011.633268

**Published:** 2012-01-31

**Authors:** André Maier, Teresa Holm, Paul Wicks, Laura Steinfurth, Peter Linke, Christoph Münch, Robert Meyer, Thomas Meyer

**Affiliations:** 1 Department of Neurology, Charité – University Hospital, Berlin, Germany; 2 Patientslikeme.com Inc. Research & Development, Cambridge, Massachusetts, USA

**Keywords:** Amyotrophic lateral sclerosis, ALS Functional Rating Scale, ALSFRS-R, online self-assessment, patient reported outcomes

## Abstract

Self-assessment of symptom progression in chronic diseases is of increasing importance in clinical research, patient management and specialized outpatient care. Against this background, we developed a secure internet platform (ALShome.de) that allows online assessment of the revised ALS Functional Rating Scale (ALSFRS-R) and other established self-assessment questionnaires. We developed a secure and closed internet portal to assess patient reported outcomes. In a prospective, controlled and stratified study, patients conducted a web-based self-assessment of ALSFRS-R compared to on-site assessment. On-site and online assessments were compared at baseline (*n* = 127) and after 3.5 months (*n* = 81, 64%). Results showed that correlation between on-site evaluation and online testing of ALSFRS-R was highly significant (*r* = 0.96; *p* < 0.001). The agreement of both capturing methods (online vs. on-site) was excellent (mean interval, 8.8 days). The adherence to online rating was high; 75% of patients tested on-site completed a follow-up online visit (mean 3.5 months, SD 1.7). We conclude that online self-assessment of ALS severity complements the well-established face-to-face application of the ALSFRS-R during on-site visits. The results of our study support the use of online administration of ALSFRS-R within clinical trials and for managing the care of ALS patients.

## Introduction

The ALS Functional Rating Scale (ALSFRS) is a validated, clinician-administered instrument for assessment in the domains of gross and fine motor function, bulbar symptoms and breathing ability in patients with ALS (1). The score reflects deterioration of function in the natural course of ALS but may have lower sensitivity in advanced disease stages (2,3). The scale was developed primarily to assess outcomes in pharmaceutical clinical trials and does not rely upon physical examinations or instruments (1,4). An initial imbalance within the scale that minimized the importance of respiratory function was rectified by a revision (ALS Functioning Rating Scale, revised (ALSFRS-R)) to incorporate respiratory symptoms and the need for ventilation (5). When administered as an interview, the ALSFRS-R shows a high inter-rater and intra-rater reliability (6,7) and can be reliably administered over the telephone (7-9).

Since its first application in 1995, the score has increasingly been used as a primary or secondary endpoint in multi-centre placebo-controlled trials (10-13). Beyond the established role of the ALSFRS-R in a trial setting there has been a growing use of the score for the assessment of ALS symptoms during the course of the disease and for clinical decision-making (14). Migration of ALSFRS-R assessment from the clinical setting (in-clinic) to an online data capturing system has the potential to improve clinical encounters, provide context to patients on their progress, and evaluate their needs in their own home and at a time of their choosing rather than subject to the scheduling of their healthcare provider. Here we report the results of a prospective controlled study of ALSFRS-R in-clinic compared with online self-assessment. We hypothesized that because the self-reported ALSFRS-R has been shown to be reliable by patients, caregivers, and even over the phone, the use of online data entry would not degrade the quality of the data. We also sought to explore the time and physical burden to patients of collecting data online to establish feasibility of online monitoring methods. Because regular assessment of inevitably declining physical function might have adverse psychological consequences for patients we also sought to explore the emotional burden of participating in the site.

## Methods

### Study protocol and IT infrastructure

We designed a prospective single-centre clinical study to evaluate the feasibility and reliability of online self-assessment. The protocol encouraged clinician-confirmed patients with possible, probable or definitive ALS (El Escorial criteria) (15) to visit the website weekly over a 52-week period. Each patient determined a week day on which to perform his or her weekly self-assessment using patient reported outcomes (PROs). In order to measure the inherent appeal of returning to the site unprompted, patients did not receive a reminder, e.g. by telephone, e-mail or text message. PROs included the ALSFRS-R, the ALS Assessment Questionnaire (ALSAQ-40) (16), the Council on Nutrition Appetite Questionnaire (CNAQ) (17) and two self-reporting assessments of dyspnoea (Borg's scale CR10, CDS) (18,19); data on these instruments will be reported separately at a later date. Subsequently, the ethics review committee and Data Security Officer approval was obtained (Ethikkomission der Charité – Universitätsmedizin Berlin, Charitéplatz 1, 10117 Berlin). We created the web-based application www.ALShome.de for data capture, a related database for storage and a content management system (CMS) for the administration and analysis of PRO measurements including the ALSFRS-R. The internet application www.ALShome.de and the CMS were developed in the programming language C Sharp (C#). Data are stored on a Microsoft® SQL database located on a secure internal server at the university data centre. The CMS allows for website management including the administration of PRO measurements, data export for further analysis and adjustment of web visit protocols at each time-interval.

### Patients and data collection

**Figure 1:**
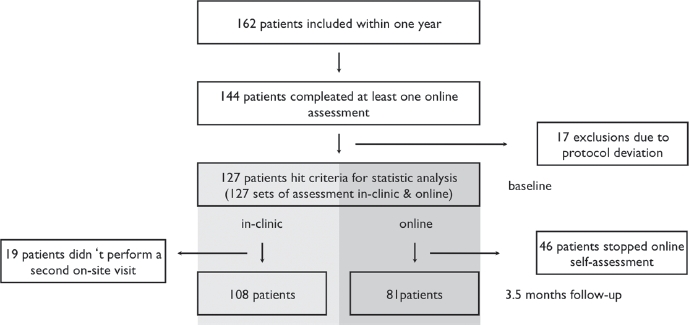
Flow chart of whole ALSFRS-R self-assessment study within one year.

Between 2 February 2010 and 2 February 2011, 162 of 443 ALS patients seen at the Department of Neurology at the Charité University Hospital of Berlin gave written consent for the trial in accordance with the Declaration of Helsinki. Lack of internet access and the refusal to participate were the most common reasons for non-attendance. One hundred and forty-four patients completed at least one online assessment (89%). We excluded 17 patients from the analysis due to protocol deviation as more than four weeks had elapsed from on-site to online assessment. In-clinic FRS assessment was collected via tablet PC administered by a nurse.Thus, in 127 patients (78%) we obtained a complete set of in-clinic assessment followed by online assessments, at baseline. The mean interval between online and on-site visits was 8.8 days (SD 6.3). The on-site follow-up data were captured from 108 patients (85% of baseline) at a visit that was, on average, 3.5 months later (SD 1.7). In 81 patients (50% of all patients included in the study) we obtained both forms of assessment at two time-points (Figure 1).

In addition, the patient was asked questions about who filled in the questionnaire (patient or caregiver), the time burden on the patient, and the physical and emotional strain of periodic online self-assessment, rated with a single-item ‘none’, ‘low’, ‘moderate’, or ‘high’ response scale.

### Data analysis

Data were analysed with IBM SPSS Statistics version 19.0.0.1 for Macintosh. Results were expressed as means (± SD) if normally distributed and medians (maximum/minimum) if distribution was non-Gaussian. Correlational analysis was performed with Spearman's rho because of the ordinal nature of the ALSFRS-R. A statistically significant difference of paired samples was analysed with a *t*-test. The difference plot method (20) by Bland and Altman was used for analysing agreement. A value of *p* < 0.01 (two-tailed) was considered significant.

## Results

**Figure 2:**
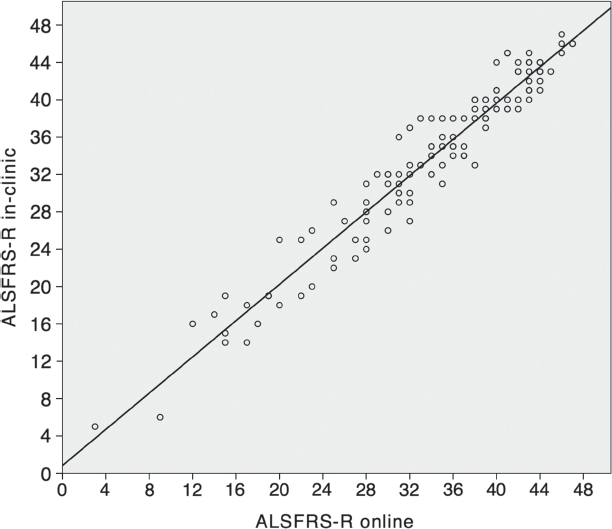
Correlation between baseline in-clinic ALSFRS-R and first online ALSFRS-R (*n* = 127, correlation coefficient = 0.96;

One hundred and twenty-seven patients (described in Table I) met the conditions for statistical analysis according to study protocol. The mean ALSFRS-R on-site at baseline was 33.6 (SD 9.1) compared to 33.8 (SD 9.1) online. The mean loss of the ALS FRS-R value per month (modified delta ALSFRS-R: 48 – ALSFRS-R at ‘time of inclusion’/duration from onset to inclusion) at baseline was 0.77 (SD 0.79), which is slightly slower progression than has been reported in other ALS studies (0.81,48 – ALSFRS-R at ‘time of diagnosis’/duration from onset to diagnosis) (21). Despite the curvilinear progression of ALS (22), this calculation method is based on a simplified linear progression model of ALS. Median time since symptom onset at baseline was 23 months. After 3.5 months the mean follow-up on-site ALSFRS-R was 31.9 (SD 8.7) compared to online 31.8 (SD 8.7). Correlation between baseline and first online ALS FRS-R was very high with a coefficient of 0.96 (*p* < 0.001) (Figure 2).

**Table I:** Characteristics of patient population (*n* =127) at baseline.

Mean age (SD; min/max)	58.0 (9.9; 35/82)
Site of onset (%)	
Limb	73
Bulbar	24
Respiratory	3
Sex (%)	M: 90 (71%)
	F: 37 (29%)
Median months since first symptom onset (min/max)	23.0 (1/141)
Mean ALSFRS-R baseline in-clinic (SD)	33.6 (9.1)
Mean loss of ALSFRS-R value per month (Delta) at baseline in-clinic (SD)	0.77 (0.79)

**Figure 3:**
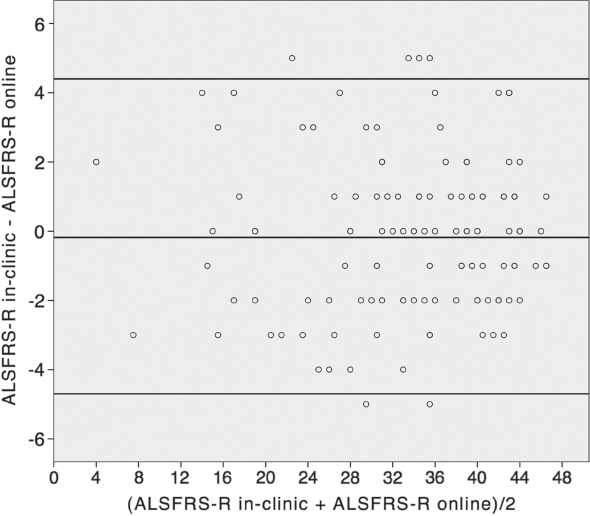
Bland-Altman plot of ALSFRS-R baseline in-clinic and first online assessment (*n* = 127, mean difference = −0.18; limits of agreement 4.4 and −4.7).

Agreement between both data-capture methods was very high (Figure 3). The distribution of differences was normal, suggesting there was no systematic directional bias to any differences. The mean difference (bias) was −0.18 and the upper and lower limits of agreement were 4.4 and −4.7, respectively. More than 95% of all pairs of measurement were within the limits of agreement.

**Figure 4:**
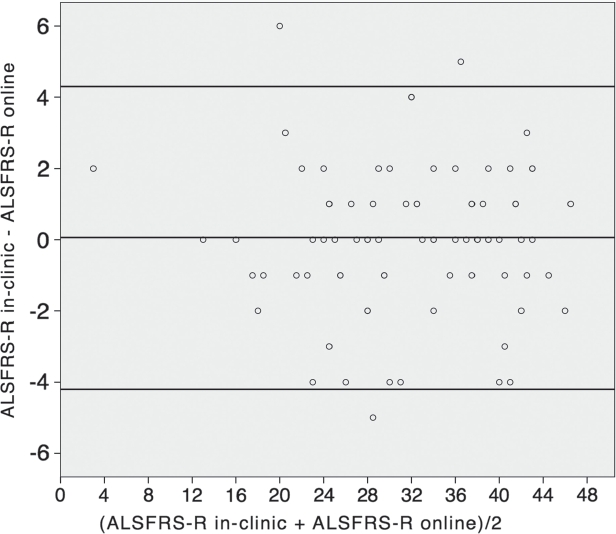
Bland-Altman plot of ALSFRS-R follow-up and closest online ALSFRS-R (*n* = 81, mean difference = 0.06; limits of agreement 4.3 and −4.2).

A similarly high degree of correlation also existed between the in-clinic follow-up visit and the closest online ALSFRS-R to this visit (0.965; *p* < 0.001; *n* = 81). In the Bland-Altman plot for follow-up visits (Figure 4), the mean difference was 0.06 and upper and lower limits of agreement were 4.3 and −4.2. Again, more than 95% of the data were within the limits of agreement. These data also suggest very good agreement in follow-ups.

Based on the additional question “who filled in the questionnaire", 22.4% of online surveys were completed by a caregiver. There was a high correlation between the self-administered baseline ALSFRS-R data and those of the two online groups, with values of 0.95 for the self-administered group (*n* = 86) and 0.92 for the caregiver-assisted group (*n* = 21) with *p* <0.01. With the Bland-Altman method, no relevant bias was detected in the two groups (data not shown).

Eleven percent of the patients who gave written consent did not complete their online assessment at all. There was no statistically significant bias according to age, disease duration or ALSFRS-R. Only a trend can be seen in gender, as there was a higher percentage of women in the group of non-completers compared to completers (50% vs. 29%, *p* = 0.075).

Table II shows the report on time burden, physical limitation and emotional strain of online self-assessment. When interviewed about the time burden and the emotional and physical strain, more than 95% of the patients who filled in the surveys felt that they were ‘not at all’ or only ‘slightly’ affected by online self-assessment. Three months on, no significant change was observed (Table III: *t*-test, *p* 0.1).

**Table II:** Time burden, emotional and physical strain connected with online self-assessment on www.ALShome.de at baseline. *n* = 112.

Question	None	Low	Moderate	High	Total
Time burden	82/82.1%	17/15.2%	2/1.8%	1/0.9%	112/100%
Emotional strain	88/78.6%	19/17.0%	4/3.6%	1/0.9%	112/100%
Physical strain	93/83.0%	16/14.3%	2/1.8%	1/0.9%	112/100%

**Table III:** Time burden, emotional and physical strain connected with online self-assessment on www.ALShome.de at follow-up. Follow-up visit after 97.3 days (SD 51.7). *n* = 78.

Question	None	Low	Moderate	High	Total
Time burden	63/80.8%	12/15.4%	2/2.6%	1/1.3%	78/100%
Emotional strain	61/78.2%	12/15.4%	4/5.1%	1/1.3%	78/100%
Physical strain	62/79.5%	8/10.3%	5/6.4%	3/3.8%	78/100%

## Discussion

We found very high correlations between ALSFRS-R scores at two time-points administered in-clinic compared to through the internet. There was no evidence of systematic bias towards higher or lower scores online. We also found that patients did not consider online ALSFRS-R entry to be physically or emotionally burdensome, or to be time-consuming. We propose for future development that a time-span between online assessments adapted to a given patient's rate of progression could be even more efficient. Completing PROs online could be a way to complement face-to-face visits and manage care in a more personalized and needs-based way, rather than relying upon regular time-intervals such as 3- or 6-month follow-up appointments. Online PROs could also be used to improve the convenience and thereby participation in clinical trials that use the ALSFRS-R as an endpoint.

Our findings must be considered in the context of their limitations. The original ALSFRS-R was designed as a paper-based clinician interview rather than a patient self-report measure, let alone one measured through the internet. However, other studies have found no evidence that self-report (23), caregiver report, or telephone administration significantly degrades the quality of the scores. Furthermore, the nature of the measure (12 items comprising short, clear questions with well-defined anchor points for response options) means that there is very little difference in user experience between the paper-pencil method and computerized administration.The move from paper-based PROs (pPROs) to electronic PROs (ePROs) (24) has prompted a number of similar studies to establish equivalence between the two methods in various medical indications and psychometric tests (25-28). On the whole, our population was relatively early in its disease course and it is unclear how its ability to respond online, or the perceived burden of the ALSFRS-R, would be felt at later stages of the disease – it is plausible that as physical function deteriorates the physical burden would increase to the point that data entry would be difficult or impossible. However, these patients also find attendance at clinic difficult, and there are numerous adaptations available to operate a computer, such that online data collection may actually increase the representativeness of data that can be collected from clinical centres.

A further limitation was that 11% of the patients who gave written consent did not complete their online assessment at all. We could not show a systemic bias in this group, but it cannot be excluded that factors such as insufficient technical requirements or discomfort with submitting data online have influence. Finally, our population was predominantly urban, seen at a specialist centre, and located in a technologically advanced country; our findings may not generalize readily to other populations and the issue of the ‘digital divide’ should be kept in mind.

Outside clinical practice, online administration of PROs has been in use for ALS patients since 2006 at the social internet platform www.patientslikeme.com. Use of the ALSFRS-R has included an extension to the scale for more disabled patients (3) and even a clinical observational study to test the effect of lithium on ALS progression (29). However, such online assessment preceded validation that collecting outcome data was viable and valid from patients with a clinically confirmed diagnosis; this study has fulfilled this important foundational step.

An internet-based assessment of ALSFRS-R may facilitate patient follow-up in the home care environment. This methodical approach contributes to greater density and continuity in the collection of outcomes that otherwise would not be possible to achieve. At the same time, it may save time and reduce costs by integrating online data into the workflow of clinic visits. Hence, this online self-assessment is a welcome addition to an electronic Case Report Form (eCRF), the most common tool for data collection in clinical trials. Dividing the internet-submitted FRS score into its component subscores (e.g. speech, walking, arm/hand, respiratory, swallowing) could also support clinicians to estimate the progress of individual symptoms and time their interventions in a needs-based manner. For instance, the fall of the respiratory subscore beneath a critical threshold may indicate the need for timely consultation.

In general, the use of patient-reported outcomes (PRO) such as ALSFRS-R has become more widespread in recent years, particularly in trials dealing with chronic disabling diseases (30,31). In compliance with the FDA standards (32), PROs supplement objective measures or replace them, especially if there is a lack of biomarkers or surrogate endpoints as in ALS. Apart from clinical trials, PRO measurements are aimed at improving patient care by intensifying the patients' involvement and considering their perspective on the disease. A study of Patient-sLikeMe's users suggested better health literacy and communication with their healthcare providers as a result of tracking their progress with PROs (33). Our study supports the notion that online self-assessment is a practicable way to integrate the patient in managing their care.

Future research should establish and quantify the potential for resource savings and improved patient outcomes in incorporating online data collection into clinical management.
